# Contamination Characteristics, Source Apportionment, and Risk Assessment of Heavy Metals in Soil from a Legacy Open Dumpsite on the Qinghai–Xizang Plateau

**DOI:** 10.3390/toxics14090804

**Published:** 2026-09-10

**Authors:** Jiamin Ma, De’an Meng, Zhixi Zheng, Mengyuan Zeng, Xiyue Sun, Zhongzhu Zhou, Peng Zhou, Guanyi Chen, Zeng Dan

**Affiliations:** 1 School of Ecology & Environment, Xizang University, No. 10, Zangda East Road, Chengguan District, Lhasa 850000, China; mjm9045@163.com (J.M.);; 2 Key Laboratory of Plateau Environmental Engineering and Pollution Control, Xizang Autonomous Region, Xizang University, No. 10, Zangda East Road, Chengguan District, Lhasa 850000, China; 3 Key Laboratory of Biodiversity and Environment on the Qinghai-Xizang Plateau, Ministry of Education, Xizang University, Lhasa 850000, China

**Keywords:** open dumpsite, heavy metals, risk assessment, metal speciation, source apportionment

## Abstract

The Qinghai–Xizang (Formerly Tibet) Plateau, known as the Roof of the World, is an important ecological security barrier for Asia and globally; its natural environment is fragile and challenging to repair after destruction. Prior to the 21st century, waste management in China primarily relied on unregulated direct dumping. To investigate the environmental impacts of open dumpsites in high-altitude regions, this study focused on a representative dumpsite in the northern Xizang region. The aim was to characterize the occurrence and distribution patterns of heavy metals (HMs) in its surrounding soil, conduct source apportionment, and perform comprehensive risk assessment. The results showed low levels of mercury (Hg) and excessive levels of all other HMs. In addition, all HMs had the highest content in the residual fraction and the lowest content in the weak-acid fraction, which is less bioavailable. The Absolute Principal Component Score–Multiple Linear Regression (APCS-MLR) and Positive Matrix Factorization (PMF) results showed that the dumpsite contributed the most to cadmium (Cd); agricultural sources contributed the most to zinc (Zn); Hg mainly came from atmospheric transport and traffic sources; Pb was mainly derived from traffic and natural sources; and copper (Cu), nickel (Ni), chromium (Cr), and arsenic (As) mainly came from the dumpsite and natural sources. In addition, this research found that this dumpsite poses a relatively high HM risk to human health; oral and respiratory routes are the main non-carcinogenic routes, As and Cr contribute more to the adult and child groups, and As contributes the most to carcinogenic risk. The calculation results of the pollution assessment method indicate that the pollution of the natural environment from this dumpsite is at a low level, and immediate remediation measures are urgently needed to achieve site decontamination.

## 1. Introduction

The phenomenon of “waste cities” is growing due to rapid societal and economic development [1]. According to the World Bank, approximately 20.16 million tons of municipal solid waste (MSW) is generated annually worldwide, of which at least 33% undergoes inadequate environmentally sound management [2]. Currently, there are four primary means of MSW disposal: sanitary landfilling, incineration for power generation, composting and pyrolysis. MSW disposal has evolved through three phases, moving from open dumpsites to sanitary landfill, and finally to an integrated approach where incineration is dominant and landfilling serves as a supplement. However, there are still many open dumpsites that have not been properly treated, which has led to significant harm to the surrounding environment [3].

Due to the lack of standardized seepage and leakage control measures, leachate from open dumpsites seeps directly into the soil and migrates to nearby water bodies, and the high concentrations of heavy metals (HMs) and bacterial viruses in the leachate result in severe contamination of the surrounding soil and water [4]. Furthermore, the malodorous gases produced by open dumpsites deteriorate the living environments of nearby households, endangering people’s physical and mental health or causing diseases and significant nuisance to the population. Recent monitoring results indicate a significant increasing trend in pollution levels at the Łubna dumpsite, Poland [5]. Nannoni’s study of an Italian dumpsite shows that the accumulation of HMs reduces plant photosynthesis efficiency [6]. Jain’s study of a dumpsite in Florida, USA, reveals significant exceedances of HM levels [7].

As the core region of the Qinghai–Xizang Plateau, Xizang features high-altitude terrain and unique natural conditions, as well as being rich in animal, plant, water, and mineral resources, making it a crucial ecological security barrier in Asia and worldwide. Between 2012 and 2022, the amount of MSW generated in Xizang increased from 256,000 tons to 622,000 tons. Meanwhile, more than 70 MSW landfills and one MSW incineration power plant have been established in Xizang so far [8]. Numerous studies have been undertaken on formal sanitary landfills in Xizang; for instance, Zhou’s study on the groundwater around the Lhasa sanitary landfill indicated that Cr^6+^ in the water poses a high cancer risk [9]. Dan’s study of Bangor county landfill indicated a moderate level of ecological risk [10]. Zhou’s study of the Shannan sanitary landfill found that its overall potential risk factor was of medium pollution hazard level [11]. However, research on open dumpsites remains limited because Xizang is sparsely populated, and before the establishment of sanitary landfills most waste was primarily disposed of in direct piles. While there has been an increase in waste scale, the open dumpsites in some remote townships have still not been properly managed yet [12]. Due to inadequate protective measures, these open dumpsites pose prominent ecological and environmental problems, mainly including unreasonable land occupation, ecological damage, malodorous pollution, breeding of mosquitoes, flies and rodents, and soil–water contamination from leachate infiltration [13,14,15].

Against this research backdrop, a large-scale, long-lived, environmentally significant open dumpsite in northern Xizang was chosen as the research object for this study. The study objectives are threefold: (1) to characterize the contents, horizontal and vertical distribution, and chemical speciation of HMs in soils surrounding a typical legacy open dumpsite in northern Xizang; (2) to quantitatively apportion the sources of these heavy metals using two complementary receptor models, Absolute Principal Component Score–Multiple Linear Regression (APCS-MLR) and Positive Matrix Factorization (PMF); and (3) to comprehensively evaluate the potential ecological risks and human health risks (including carcinogenic and non-carcinogenic risks) posed by the dumpsite to the surrounding environment and local population. This research addresses the knowledge gap regarding open dumpsites in the Qinghai–Xizang Plateau, provides scientific data to support subsequent remediation efforts by relevant authorities, and serves as a reference for scholars in related fields.

## 2. Materials and Methods

### 2.1. Study Area and Sample Collection

**Figure 1:**
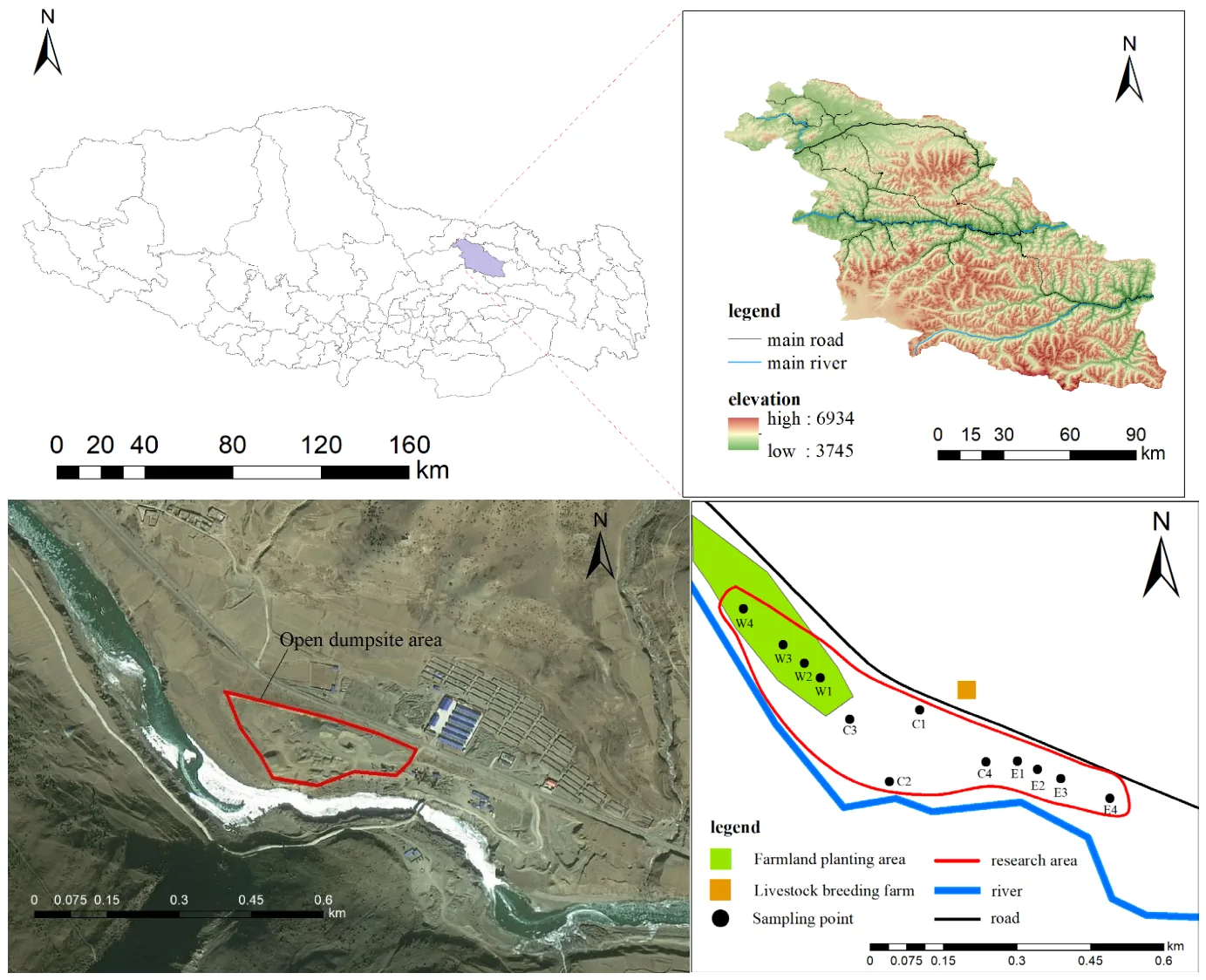
Sampling points of research area and the open dumpsite.

The study area is situated in the northeastern part of Xizang (approximately 31°28′–31°52′ N, 93°30′–94°05′ E), characterized by high mountain valleys, an average elevation of more than 4000 m, and a plateau sub-cold monsoon semi-humid climate, with an average annual temperature of 2.9 °C and annual precipitation ranging from 580~650 mm. Soils in the region are mainly Cambisols and Leptosols, according to the World Reference Base for Soil Resources (WRB), developed from diverse lithologies including Triassic, Jurassic, Cretaceous, and Quaternary deposits. The regional geology is characterized by complex tectonic transition zones between the Tanggula Mountains in the north and the Gangdise–Nyainqentanglha Mountains in the south. This open dumpsite serves as the primary waste disposal site for local residents, is located in the lower part of the county, functioning as a valley-type open dumpsite, and has been in operation for nearly 20 years, occupying an area of 4.24 hectares as of today. The site was selected as representative because it is the largest and oldest unregulated dumpsite in the region, with no engineered liners or leachate collection systems, and is situated adjacent to both agricultural land and the Nujiang River—making it an ideal case for assessing the environmental impacts of legacy waste disposal in high-altitude, ecologically sensitive areas. The waste deposited at the site is primarily domestic waste, composed mainly of plastics, textiles, paper, metals, glass, and kitchen waste, with a significant proportion of combustible materials. The north side of the open dumpsite borders a highway, beyond which lies a livestock farming base. Its southern boundary is adjacent to the Nujiang River, with a protective fence installed along the riverbank to prevent waste from entering the waterway. The elevation difference across the site ranges from 20 to 70 m. Woodlands border the eastern flank, while agricultural fields are distributed to the west. The terrain is generally flat in the northern and southern portions but slopes gently from east to west, with the downstream side of the dumpsite located on the western side. The location of the open dumpsite is shown in Figure 1.

### 2.2. Sample Pretreatment and Heavy Metal Detection

Soil samples were collected in August 2023 during a single field campaign. The sampling design was established based on the internal and peripheral environmental conditions of the dumpsite. Specifically, four sampling points (C1–C4) were set inside the site, with one point placed in each of the four cardinal directions (north, south, east, and west) to capture potential heterogeneity within the waste-affected area. Outside the site, along the eastern and western transects, samples were collected at distances of 10 m, 50 m, 100 m, and 200 m from the dumpsite boundary (designated E1–E4 and W1–W4, respectively). At each sampling point, five sub-samples were collected within a 5 m radius and homogenized to form one composite sample (approximately 1 kg), ensuring spatial representativeness while minimizing local-scale variability. Additionally, to investigate vertical migration, soil samples were collected at four depth intervals (0–10, 10–20, 20–30, and 30–50 cm) at selected locations. At a depth of approximately 50 cm, substantial bedrock was encountered during excavation. This is primarily attributed to the relatively thin soil layer on the Qinghai–Xizang Plateau, which is a consequence of its Cenozoic soil-forming environment. Under these conditions, deeper soil sampling was not feasible. Background samples were collected 5 km away from the dumpsite, upwind and upstream, to represent local geochemical baseline conditions. The detailed sampling locations are illustrated in Figure 1.

The collected soil samples were sieved through a 1 mm nylon sieve after removing large debris. Soil moisture content was measured according to the Soil—Determination of dry matter and water content—Gravimetric method (HJ 613-2011 [16]). Each sample was then air-dried at room temperature in the laboratory and labeled with its corresponding sample number. The numbering was used solely for identification and traceability and did not affect the drying process. After drying, the soil samples were ground to pass a 200-mesh nylon sieve. Soil pH was measured according to the Soil—Determination of pH—Potentiometry (HJ 962-2018 [17]), and organic matter content was determined according to the Solid waste—Determination of organic matter—Ignition loss method (HJ 761-2015 [18]).

For total heavy metal concentrations, soil samples were digested with aqua regia following [Soil and sediment—Determination of aqua regia extracts of 12 metal elements—Inductively coupled plasma mass spectrometry] (HJ 803-2016 [19]). The digests were analyzed using inductively coupled plasma mass spectrometry (ICP-MS, Thermo Fisher Scientific iCAP RQ, Waltham, MA, USA) for the determination of cadmium (Cd), zinc (Zn), copper (Cu), nickel (Ni), chromium (Cr), lead (Pb) and arsenic (As). Mercury (Hg) was determined separately using a direct mercury analyzer (DMA-80, Milestone, Sorisole, Italy) according to the Soil and sediment—Determination of total mercury—Catalytic pyrolysis-cold atomic absorption spectrophotometry standard (HJ 923-2017 [20]).

For heavy metal speciation analysis, the weak-acid-extractable, reducible, oxidizable, and residual fractions of Cd, Zn, Cu, Ni, Cr, Pb, As, and Hg were sequentially extracted following the Soil and sediment—Sequential extraction procedure of speciation of 13 trace elements standard (GB/T 25282-2010 [21]). This standard specifies the extraction conditions and procedures for five operationally defined fractions: weak-acid-extractable, reducible, oxidizable, residual, and water-soluble fractions. In this study, the water-soluble fraction was not analyzed, as preliminary tests showed concentrations below detection limits for all target elements. All extractions were performed in triplicate, and reagent blanks were processed in parallel to ensure quality control.

### 2.3. Statistical and Analytical Methods

#### 2.3.1. Methods for Analyzing the Sources of Heavy Metals

The Absolute Principal Component–Multiple Linear Regression Analysis (APCS-MLR) method was calculated for each sample by subtracting the factor scores of the “zero-concentration sample” from the principal component scores derived via factor analysis. In the APCS-MLR procedure, the “zero-concentration sample” is not a real observation but serves to rescale the principal component factor scores to absolute values, enabling subsequent multiple linear regression to estimate the source contributions for each sampling point. Using the APCS as the independent variable and the target contaminant concentrations as the dependent variable, a multiple linear regression (MLR) model was established. The resulting regression coefficients allow for the transformation of APCS into estimates representing the concentration contribution of each identified pollution source to every individual sample. The formula is as follows.

The regression coefficient $a_{pi}$ represents the contribution of known pollution sources. The contribution rate is calculated using the following formula:

(1) $$PC_{i}=\frac{\sum_{p=1}^{p}\left|\left(a_{pi}\times {\bar{APCS}}_{pi}\right)\right|}{\sum_{p=1}^{p}\left|\left(a_{pi}\times {\bar{APCS}}_{pi}\right)\right|+\left|b_{i0}\right|}$$

The constant term b_i0_ is considered to be the unknown source contribution and the contribution rate is calculated using the following formula:

(2) $$PC_{i}=\frac{\left|b_{i0}\right|}{\sum_{p=1}^{p}\left|\left(a_{pi}\times {\bar{APCS}}_{pi}\right)\right|+\left|b_{i0}\right|}$$

The Positive Matrix Factorization (PMF) receptor model is a mathematical method employed to quantitatively analyze the source composition of samples. It achieves this by decomposing the input data matrix to ascertain the contribution rates of potential pollution sources to each sample. A given dataset can be considered as a data matrix X of i, where i number of samples and j chemical substances are measured with uncertainty u. The basic equation is

(3) $$X_{ij}=e_{ij}+\sum_{k=1}^{p}g_{ik}f_{kj}$$

#### 2.3.2. Methods for Analyzing the Status of Heavy Metal Pollution

HM pollution was analyzed using the single factor index method (PI), the geo-accumulation index (I_geo_) method, and the comprehensive pollution load index (PLI) method, with specific formulas as follows:

(4) $$P_{i}=\frac{C_{i}}{B_{i}}$$

(5) $$I_{geoi}=\log_{2}(\frac{C_{i}}{1{.5B}_{i}})$$

(6) $$PLI=\sqrt[{n}]{Pi_{1}\times Pi_{2}\times Pi_{3}\times \cdots Pi_{n}}$$

where $P_{i}$ is the single factor pollution index of heavy metal i, $C_{i}$ is the measured value of heavy metal i, and $B_{i}$ is the reference value of heavy metal i. The background value of soil collected in the study area is used, $I_{geoi}$ is the geo-accumulation contamination index of heavy metal i, and PLI is the comprehensive pollution load index of a point.

#### 2.3.3. Methods for Analyzing Heavy Metal Accumulation Patterns

The Risk Assessment Coding (RAC) method utilizes the total mass fraction of the weak-acid fraction F1 to evaluate the magnitude of the migration capacity of the HMs, while the Khan-Risk-Synthesis-Potential (KRSP) is used to analyze the bioavailability of the HMs. The primary phase refers to the portion of the more stable HM forms produced by weathering of soil parent minerals, i.e., the residual fraction F4 as defined in this study, and the secondary phase refers to the available fraction in this study, including the weak-acid fraction F1, the oxidizable fraction F2, and the reducible fraction F3. The specific formula is as follows:

(7) $$RAC_{i}=\frac{C_{F_{1}}}{C_{T}}$$

(8) $$KRSP=\frac{C_{\sec}}{C_{prim}}\times 100\%$$

Here, the $RAC_{i}$ index of heavy metal i is presented, $C_{F_{1}}$ is the content of heavy metal in weak-acid fraction, and $C_{T}$ is the total content of heavy metal. $C_{\sec}$ is the mass fraction of soil HMs in the secondary phase, and $C_{prim}$ is the mass fraction of soil HMs in the primary phase, both in mg kg^−1^.

#### 2.3.4. Heavy Metal Risk Assessment Methodology

(1) Potential ecological pollution index

The potential ecological pollution risk index (PERI) method is a comprehensive evaluation method using the environmental toxicity effects of different HMs on the ecological environment, characterizing the ecotoxicity effects of compound pollution caused by a variety of HMs. The risk level of individual soil heavy metal pollution is divided into 5 levels, ranging from low risk to severe ecological risk, and the comprehensive potential ecological risk hazard index RI is divided into 4 levels, from low risk to severe risk. The classification criteria and toxicity response coefficients of HMs are presented in Table S1 in the attachment. The calculation formulas are given in Equations (9) and (10).

(9) $$E_{r}^{i}=P_{i}\times T_{r}^{i}$$

(10) $$RI=\sum_{i=1}^{n}E_{r}^{i}$$

where $E_{r}^{i}$ is the potential risk index of heavy metal i, $P_{i}$ is the single factor pollution index of heavy metal i, $T_{r}^{i}$ is the toxicity factor of heavy metal, and RI is the total potential ecological pollution risk index.

(2) Human health risk assessment methodology

The human intake of HM components mainly involves respiratory, dermal, and oral routes, among others. This study utilizes parameters for both adult and child populations (refer to Table S2), and the calculation formulas are as follows:

(11) $$\mathrm{Non}-\mathrm{carcinogenic}\;\mathrm{risk}\;\mathrm{index}:\;HQ=\frac{ADI}{RfD}$$

Total non-carcinogenic risk index:

(12) $$HI=\sum_{i=1}^{m}\sum_{j=1}^{n}HQ_{ij}=\sum_{i=1}^{m}\sum_{j=1}^{n}(\frac{ADI}{RfD}{)}_{ij}$$

Cancer risk:

(13) R=LADD×CSF

Total cancer risk:

(14) $$TR=\sum_{i=1}^{m}\sum_{j=1}^{n}R_{ij}=\sum_{i=1}^{m}\sum_{j=1}^{n}{\left(LADD\times CSF\right)}_{ij}$$

The carcinogenic risk is calculated from the average daily intake (ADI), the lifetime average daily dose (LADD) and the carcinogenic slope factor (CSF). The reference doses and slope factors of the elements used in the study were chosen according to the classification system of the International Agency for Research on Cancer (IARC) and the World Health Organization (WHO), as well as by referring to the reference doses and carcinogenicity slope factors in the relevant studies (refer to Table S3).

## 3. Results and Discussion

### 3.1. Soil Physical and Chemical Properties

**Figure 2:**
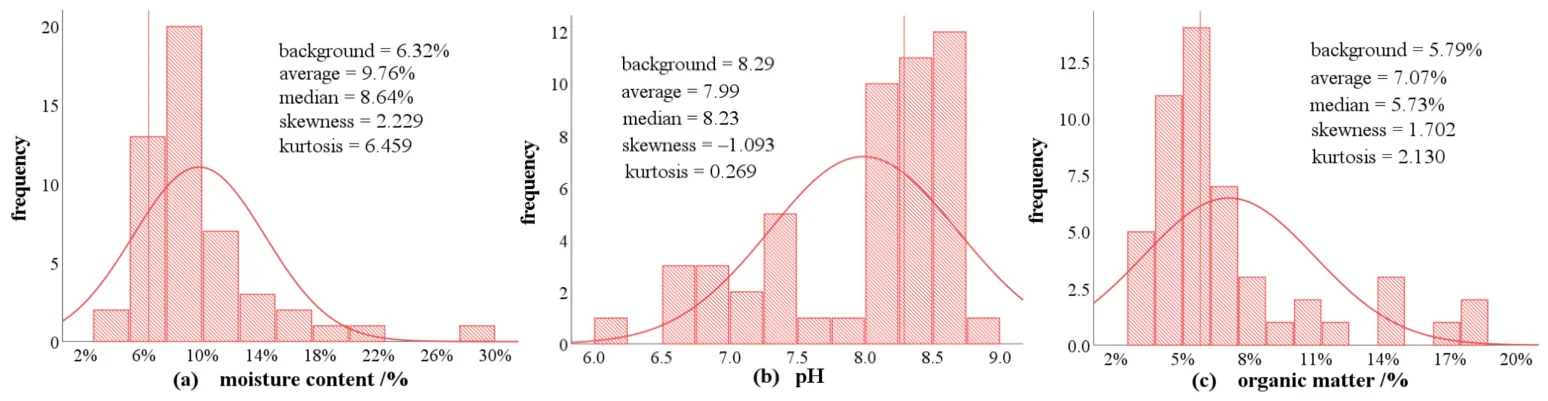
Frequency distribution and Gaussian fitting diagram of soil physical and chemical properties.

Frequency distribution analysis was conducted on soil moisture content, pH, and organic matter content, with Gaussian fitting curves generated to model the distributions. The results are presented in Figure 2. The soil water content of the dumpsite ranged from 3.15% to 28.75%, with an average value of 10.06%; the water content of the surface soil was higher, with an average value of 13.52%, and the water content decreased with increasing soil depth. The pH ranged from 6.01 to 8.77, with an average value of 7.99. Alkaline samples accounted for 86% of the samples, and the organic matter content of the soil ranged from 3.02% to 18.43%, with an average value of 7.07%. Additionally, 42% of the samples exceeded the background value of the organic matter content, and the point samples with higher contents were mainly concentrated in the surface soil on the east and west sides of the dumpsite outside. The soil background of the Qinghai–Xizang Plateau is alkaline [22]. The points of low pH in the study area were essentially all downstream of the dumpsite, in the middle of the farmland to the west, and may be caused by fertilizer application [23].

### 3.2. Analysis of Soil Heavy Metal Content

#### 3.2.1. Content Characteristics

The distribution of HM content is characterized in Figure S1. Hg was detected in only a few samples, with concentrations in the remaining samples falling below the detection limit, without forming an obvious box. Most HMs exceeded the background value but did not exceed the risk screening value; only Cd and Zn existed in 1 or 3 samples exceeding the screening value. The background level of As was higher than the screening level, indicating that the high As content in the parent soil in this study area is due to the unique conditions of the parent soil [24]. The HM concentrations in this open dumpsite ranged from 0.74 to 1.52 times those of Lhasa’s sanitary landfill [8], 1.43 to 3.33 times those of Jiangsu Province [25], 1.01 to 5.53 times those of Sichuan Province [26], and 1.46 to 5.34 times those of Gansu Province [27]. Comparative analysis against referenced studies on sanitary landfills across China indicated significantly elevated contamination levels at this northern Xizang open dumpsite.

#### 3.2.2. Horizontal Distribution

**Figure 3:**
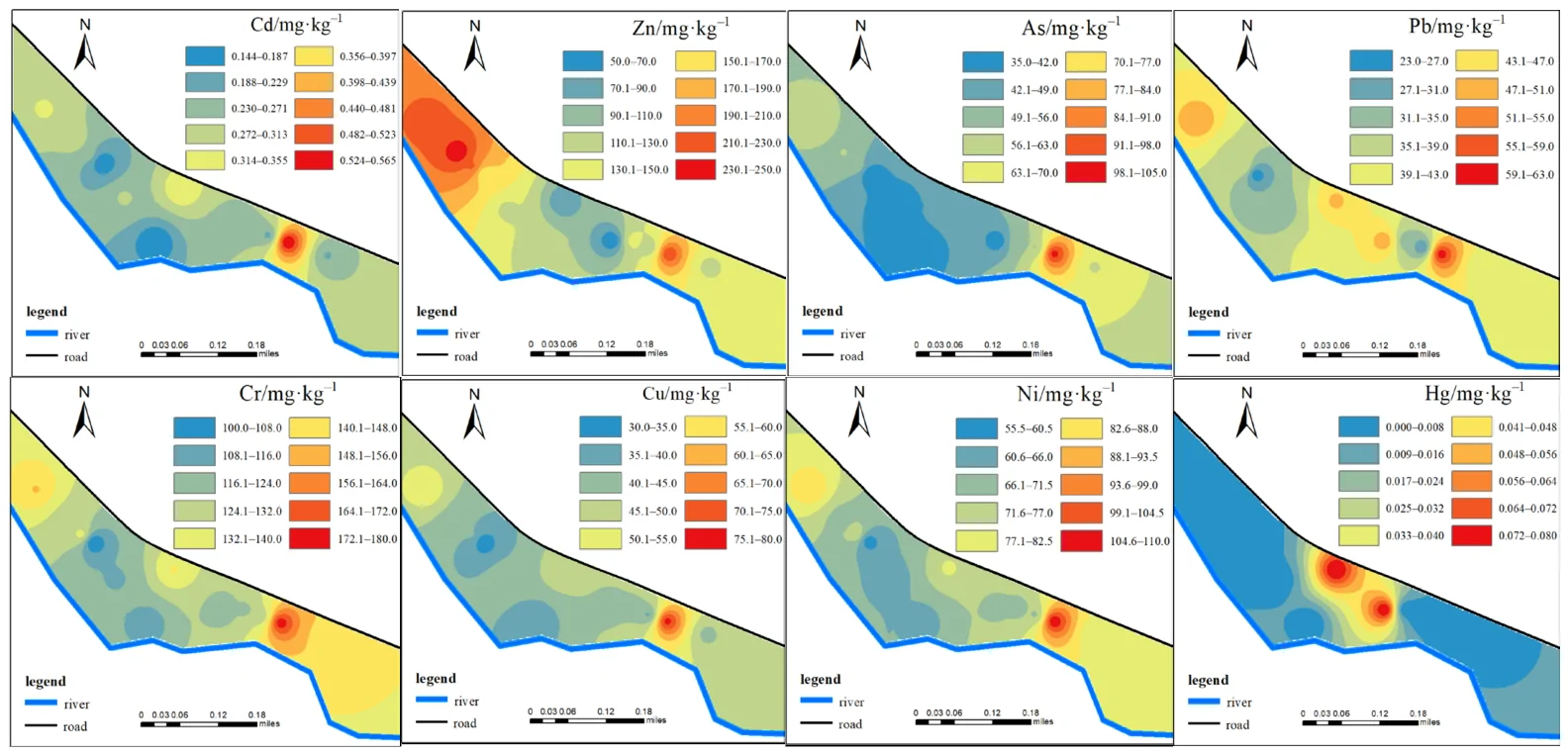
Distribution map of HM content levels in the soil of the study area.

The Inverse Distance Weighting (IDW) method was used to analyze the horizontal distribution characteristics of HMs, and the results are shown in Figure 3. The distributions of Cd, As, Cr, Cu, and Ni exhibited similar patterns. The HM content within the dumpsite was lower than that outside the site area, concentrations in the eastern external area surpassed those in the western external area, and there was a one-point high value area located in the east side of point E3. Field investigations revealed that this spatial pattern is attributable to the site’s recent implementation of closure management and disinfection procedures. During this period, domestic waste was temporarily stored at point E3, resulting in elevated HM concentrations detected at this sampling point. Zn concentrations outside the dumpsite boundary exceeded those within, with higher levels prevalent in the western external area compared to the east, and the highest value at point W3 due to fertilizer application [28]. Hg was detected solely at points within the eastern and northern areas of the site; concentrations at all other locations fell below the detection limit. This is because the north side was adjacent to the highway and the east side was the entrance to the site for removal trucks, and vehicle exhaust was found to be a significant source of Hg [29,30]. The result illustrates that the HMs in the study area have undergone significant outward diffusive transport in horizontal distribution.

#### 3.2.3. Vertical Distribution

**Figure 4:**
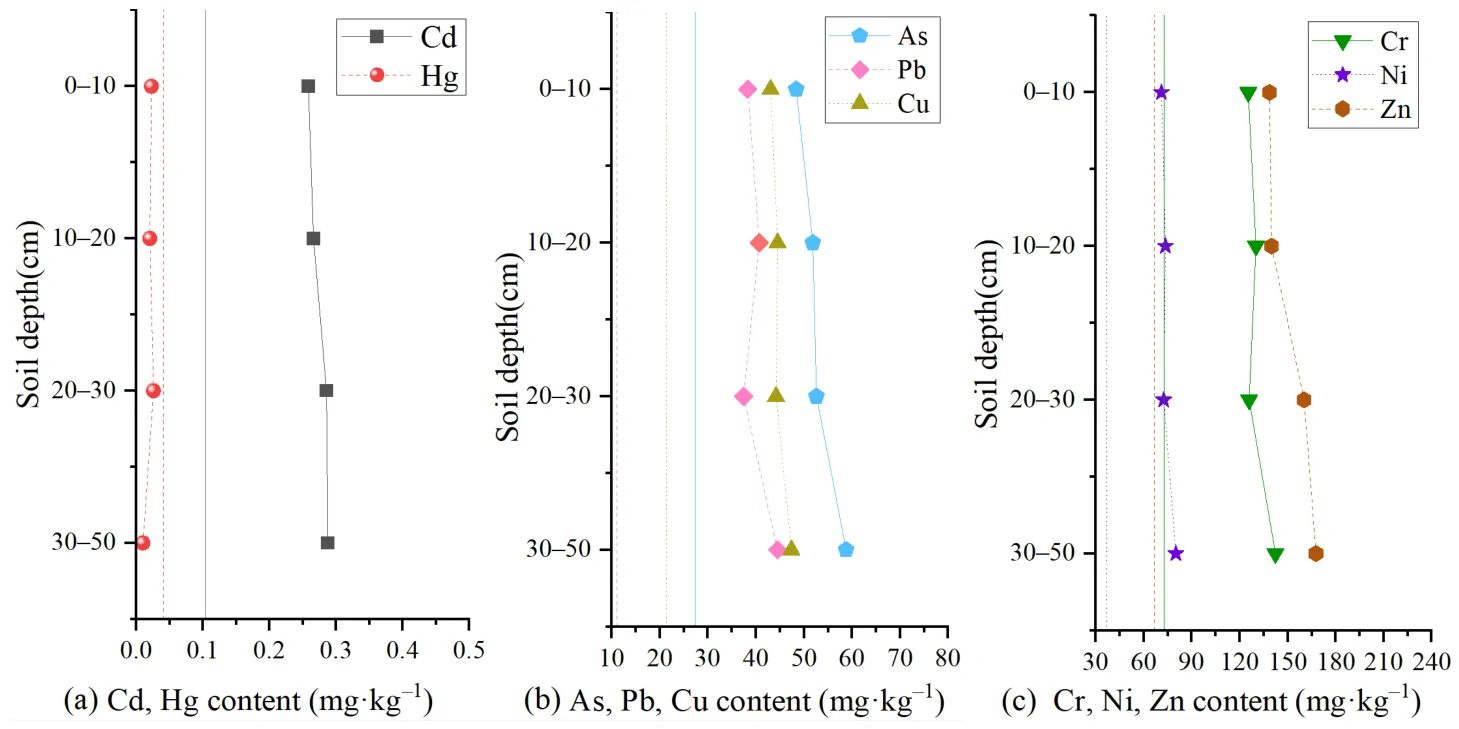
Depth distribution maps of heavy metal content in the soil.

The vertical distribution characteristics of HMs are illustrated in Figure 4, with vertical reference lines added to the soil background values. Since Hg was not detected in most of the sampling points, it was excluded from discussion. The contents of Ni, Zn, Cd, As increased with the depth of soil, and the concentrations of Cr, Ni, and Pb exhibited a pattern of initially increasing, followed by a slight decrease, and then a subsequent rise. All the HMs reached the maximum at a soil depth of 30–50 cm, and all HMs at different soil depths exceeded the background values of the soil in the study area, indicating that the downward migration of HMs in the study area occurred in the vertical direction, consistent with the findings reported by Ruan and Tang [31,32].

#### 3.2.4. Speciation Analysis

The results of the HM speciation analysis are shown in Figure S2. The residue fraction had the highest levels of all elements, and the weak-acid fraction had the lowest levels. The weak-acid fraction of Cd was the highest among the seven HMs, reaching up to 16.23%, followed by Zn and Ni, with an average of 2.66% and 2.31%, respectively. The results showed that Cd is most susceptible to migratory transformation within the study area; it was also more easily absorbed and utilized by plants and was the most environmentally toxic, consistent with previous findings [33]. Zn and Ni were the next-most-active elements, while the other four elements were less active. The main reason for this result was environmental pollution from the dumpsite, which increased the activity of HMs [34,35]. Specific analyses of HM speciation revealed that the bioavailable fraction of Cd constituted 20.99% of total content in Guangxi Province [36]. Results from Guizhou province indicated that Cd primarily existed in acid-soluble and residual forms, while Cr, Pb and Mn were dominated by reducible fractions, with Zn and Cu predominantly residual [37]. The results for Jiangsu province demonstrated that Cd’s reactive phases accounted for 31.9% [38]. Studies on Gansu province studies showed acid-soluble metal concentrations following the order Cd > Pb > Zn > Cu [39]. Comparatively, the open dumpsite exhibited lower HM reactivity, which was probably due to the alkaline soil background of the whole Qinghai–Xizang Plateau, which suppressed the reactivity of HMs to a significant extent [22,40].

The reducible fraction contents of Cd, Zn, and As were higher, and the sample points with higher concentrations were mainly located near the dumpsite and on the west side of the site. There were agricultural fields on the west side of the site with abundant plants, which provided a good reaction site for microbial activities, consistent with the findings reported by Wang [41]. Microbial activities altered the redox conditions of the soil, which in turn led to the conversion of HMs from their organically complexed forms to forms in species more readily assimilated by plants [42]. Similar reactions were seen underneath the waste pile. In conjunction with Section 3.3.1, the bioavailable fractions of these three elements were significantly and positively correlated with moisture content and organic matter. This suggests that the oxidizable forms of HMs are more readily taken up by plants at these points, resulting in higher levels of HMs in the reducible fraction of the soil. The oxidizable contents of Pb, Cr, Cu, and Ni were higher, and the sample points with higher content were mainly located on the eastern and northern sides of the site. The exhaust gas produced by vehicles contributed to the combination of sulfides, organic matter, and HMs, resulting in a higher oxidizable content [43]. Previously analyzed point E3, with abnormally high levels of HMs, had relatively high secondary phase content compared to all other sites. This indicated that although the waste here was only temporarily stored and then transported to the site, the HMs were converted to the bioavailable phase and accelerated, then released into the soil during the pre-reaction phase of the predominantly aerobic decomposition of the waste dump [44]. This led to a sharp increase in the HM content and made the soil environment more susceptible to pollution. Therefore, seepage and leakage prevention treatments for dumpsites can better control the outward migration of HMs [45].

### 3.3. Source Apportionment of Heavy Metals in the Soil

#### 3.3.1. Correlation Analysis

The Pearson correlation analysis of the soil physicochemical properties and each heavy metal is shown in Figure S3 of the attachment. Among the soil physical and chemical properties, pH and organic matter were significantly negatively correlated, with a correlation coefficient of −0.38, indicating that aerobic degradation of the dumped organics leads to the formation of organic acids, lowering soil pH [46]. Cd, As, Pb, Cr, Cu and Ni were highly correlated with each other, indicating that these HMs share the same source of contamination. Zn was also positively correlated with As, Cr and Ni, but the correlation level was low. Hg was negatively correlated with Zn and As [47], but showed no significant correlation with the remaining four species; it was hypothesized that there may be other separate sources of Zn and Hg.

The weak-acid fraction of As was significantly positively correlated with moisture content and organic matter, while the oxidizable fraction was negatively correlated with pH and positively correlated with organic matter. Under alkaline conditions, As is mainly found in the precipitated fraction with relatively low activity, whereas acidic conditions promote As mainly in the weak-acid fraction with high biotoxicity [48]. The higher the organic matter content, the more likely As is to be immobilized in an organically bound fraction, which affects its bioavailability and transport capacity [49]. The reducible fraction of Ni was positively correlated with pH and negatively correlated with organic matter. Ni tends to combine with a carbonate or ferromanganese bond under neutral and alkaline conditions, showing a higher form of reducible fraction. A deprotonation reaction occurs when the organic matter content is high, forming a stable complex with low toxicity and poor migration ability with Ni, then inhibiting the formation of reducible Ni [50].

#### 3.3.2. APCS-MLR Method Analysis

The Kaiser–Meyer–Olkin (KMO) value for the APCS-MLR method was 0.831, which is greater than 0.6, and Bartlett’s test of sphericity significance gave a result of 0.000, which is less than the significance level of 0.05. The value of the approximate chi-square statistic was 444.936, which indicates that the data is suitable for APCS-MLR. After Kaiser rotation, three principal component factors were obtained, as shown in Table S4; the cumulative total explanatory rate of the three principal components was 89.89%, explaining 60.50%, 15.58%, and 13.81% of the total variance, respectively. Factor 1 featured large loadings on Cd, As, Pb, Cr, Cu, and Ni, factor 2 showed large loadings on Hg, and factor 3 involved large loadings on Zn, all with loading coefficients greater than 0.8.

**Figure 5:**
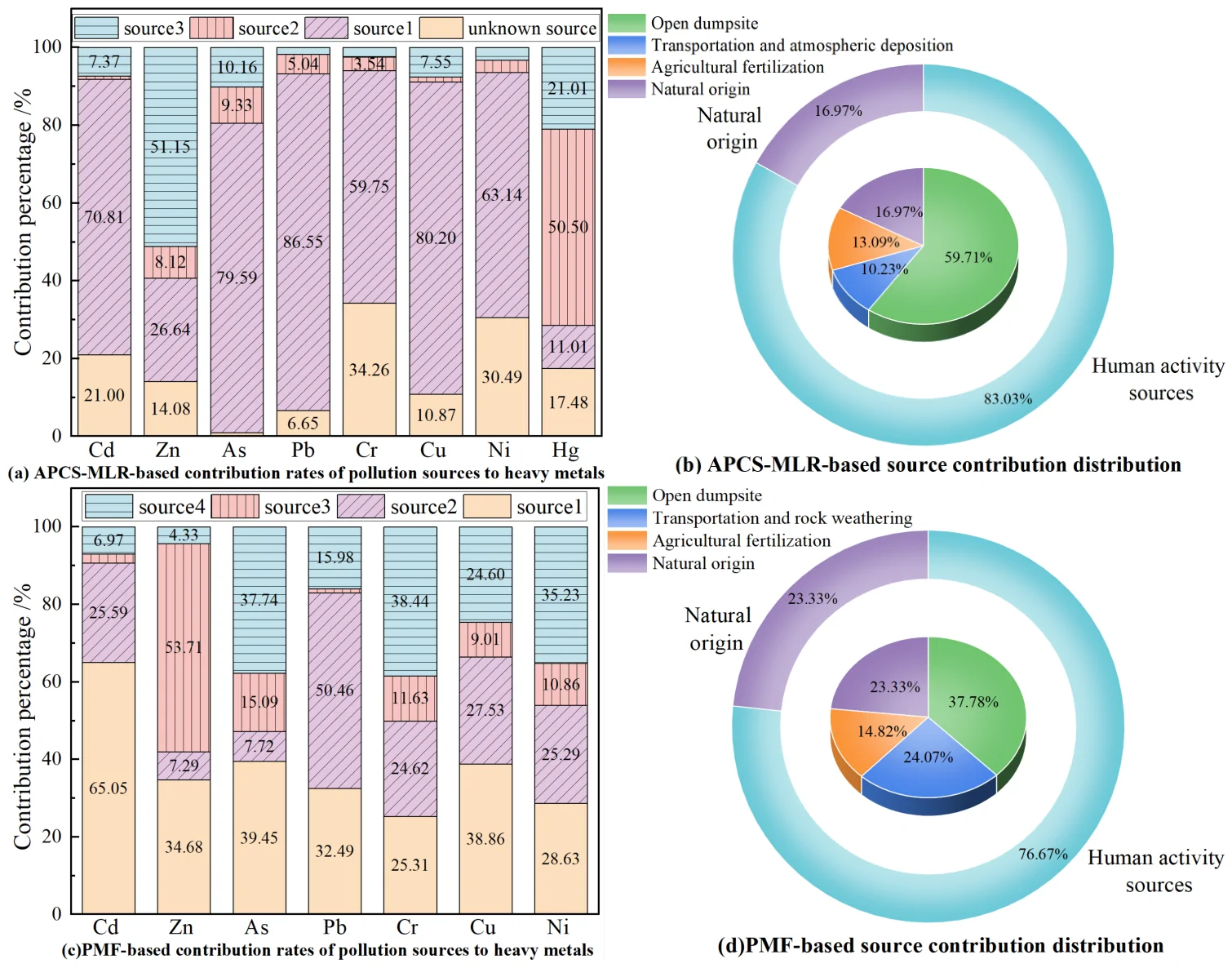
APCS-MLR and PMF source apportionment models contribute to HM pollution concentration.

The loadings of the different component factors on each element were analyzed using multiple linear regression, yielding an unknown source, and the results were plotted as shown in Figure 5a. Factor 1 contributed more to Cd, As, Pb, Cr, Cu, and Ni, and was therefore judged to be the dumpsite source. Factor 3 contributed the most to Zn, and combined with the spatial distribution of HMs, Zn was mainly distributed in the farmland on the west side of the site. It can therefore be judged that factor 3 is a source of human agricultural activities. Factor 2 contributed the most to Hg, and Hg was mainly distributed in the northeast side of the dumpsite, which was analyzed in conjunction with the fact that the overall Hg in the Qinghai–Xizang Plateau was mainly derived from atmospheric transportation and deposition [51]; thus, factor 2 was judged to be source pollution from traffic and atmospheric deposition. Notably, elevated As concentrations were observed in the collected soil background samples, attributable to the region’s unique bedrock geology and parent material. However, there was a discrepancy between the analytical results of APCS-MLR and the measured expression of As content; additional methods are needed to explain this situation.

#### 3.3.3. PMF Model Analysis

The data were modeled using EPA PMF, which identified four source factors. A random starting seed number was chosen, and 40 iterations were run. All seven elements were Strong except Hg, which was Bad, and the R^2^ for all elements was greater than 0.8, suggesting that the data are suitable for the PMF source apportionment method. At the 18th run of the data, Q_Robust_/Q_True_ was closest to 1, giving the best run; the PMF results are shown in Figure 5c. Factor 1 contributes more to Cd and Cu, which are usually associated with the electronics industry, and in the context of this study, could have been caused by untreated waste such as discarded electronics, so Factor 1 was deemed to be the dumpsite source. Factor 3 contributed most to Zn, which was an active agricultural source. Factor 2 had the highest contribution to Pb, which was usually related to the emission of automobile exhaust, while factor 2 had a high contribution to Cu, Cd and Ni, which are elements commonly found in the earth’s crust under natural conditions, so it was judged that factor 2 is a source of transportation and rock weathering [52]. Factor 4 was a major contributor to Cr, As and Ni. Combined with the high background level of As in the soil and the specific alpine and high-altitude climate of the site, it can be concluded that Factor 4 is a natural source.

A comparative plot of the source apportionment results obtained from the two distinct methodologies is presented in Figure 5b,d. The APCS-MLR results indicated that the natural sources of soil HMs in the study area accounted for 16.97% of the total, and the pollution source due to anthropogenic activities accounted for 83.03% of the total. The PMF results indicated that natural sources of soil HMs in the study area accounted for 23.33%, while anthropogenic sources contributed 76.67% [53]. It can be seen that the differences in the results of the two methods were mainly due to the different sources of contributions to As and Pb and to the peculiar soil-forming parent material conditions in the study area, resulting in higher background levels of As. However, both methods indicated that human activities are the main cause of the changes in the HM content of the soil in the study area [54].

### 3.4. Pollution Risk Assessment

#### 3.4.1. Environmental Risk Assessment

**Figure 6:**
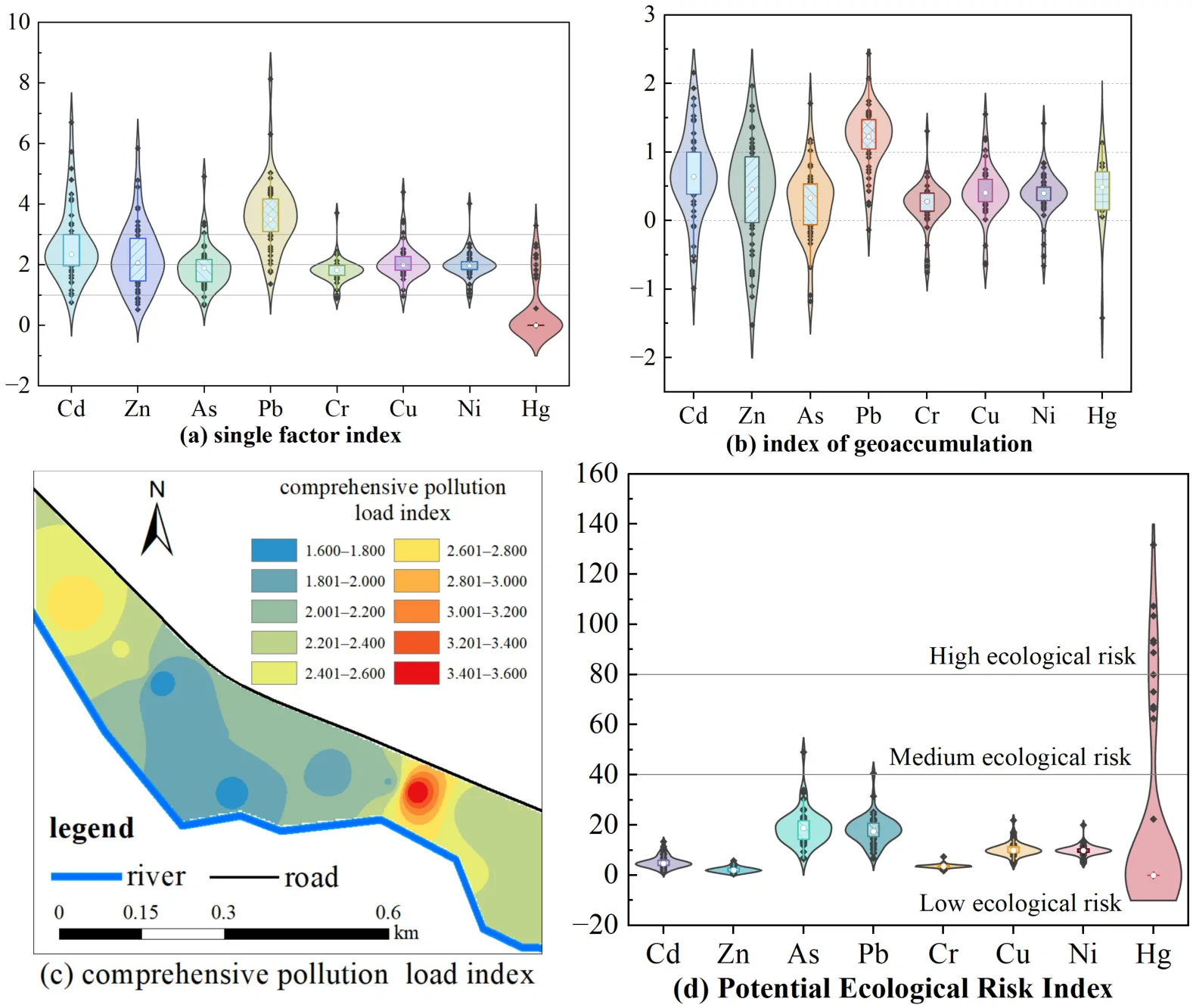
Pollution risk assessment results of soil heavy metals.

The grading standards are detailed in Table S5, and the results are presented in Figure 4. The results of the PI evaluation are shown in Figure 6a. The main distribution densities of Cd, Zn, As and Cu primarily exhibited a moderate contamination level. Cr was concentrated between 1 and 2, corresponding to a light contamination level. Ni demonstrated contamination levels spanning light to moderate, and Hg was in the pollution-free level. Pb was classified as exhibiting moderate and severe pollution and contributed the largest proportion. The results of I_geo_ are shown in Figure 6b. It can be seen that Cd, Zn, As, Cr, Cu, Ni, and Hg primarily exhibit a light pollution level, with Pb at a moderately polluted level, and two and one sampling points were at moderate levels of contamination for Pb and Cd, respectively. The most severely contaminated point was located in E3, which was consistent with the results of the previous analysis, in addition to showing a pattern of increasing with the depth of the soil, indicating that the contamination of the deeper soil layer is more severe than the surface layer. The results of the PLI are shown in Figure 6c, and was mostly distributed in light and moderate pollution levels. The total points with light pollution and those that were pollution-free amounted to 90%, and the points in the moderate or worse pollution criterion accounted for 10%. Point E3 was seriously polluted, which led to the appearance of a high-value point area, and the east side was more seriously polluted than the west. The three pollution assessment methods collectively indicate a low level of HM contamination in the soils surrounding the dumpsite.

The results of the PERI are shown in Figure 6d. The average values of the single-element PERI for all elements in the study area were below 40, which indicated a low level of ecological pollution risk, but there was one sample point which included both As and Pb at one point at the medium risk level, which was located in E3. While Hg primarily presented a low risk level of pollution, its elevated inherent ecotoxicity contributed to four sites exhibiting medium ecological risk and seven sites showing higher risk. The E_r_^i^ was accumulated to obtain RI, of which 42 samples less than 150 had low risk and 8 samples had moderate risk; these findings indicate a low overall potential ecological risk across the study area.

#### 3.4.2. Assessment of the Risk of Heavy Metal Speciation

**Figure 7:**
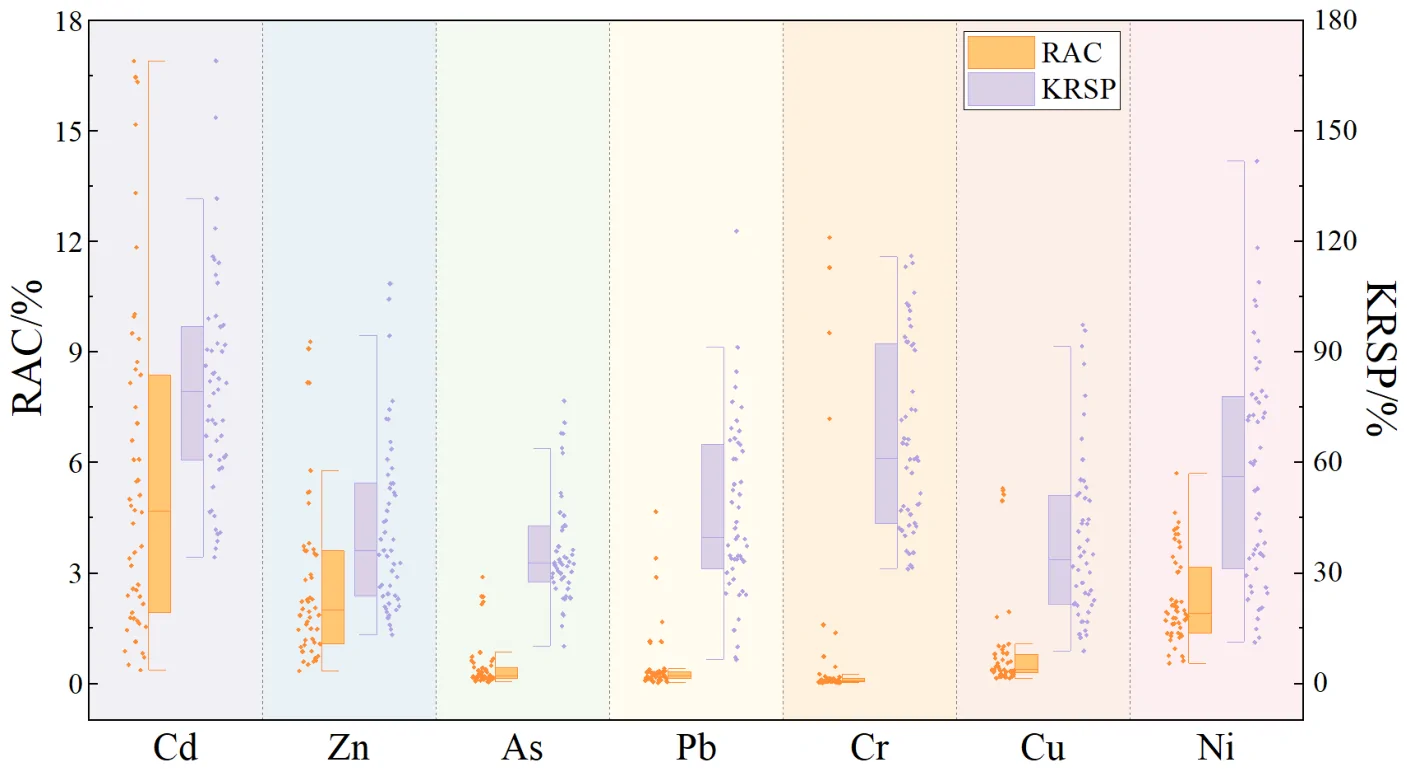
HM speciation risk assessment results.

As the Hg concentration was below the detection limit, it was excluded from the specific analysis. The grading standards are detailed in Table S6, and results are shown in Figure 7. The RAC results of Pb, Cr, Cu, and As were at no risk for more than 90% of the samples, and the RAC results for Cd, Zn, and Ni were at light risk for more than 60% of the samples and at moderate risk for only 7 samples for Cd and 2 samples for Cr, which were located in the E3 and W4 points, which are at light risk of contamination as a whole. The KRSP of Cd, Zn, Pb, Cr, and Ni involved nine, two, one, seven, and five samples, respectively, at slightly polluted levels, and the remaining samples presented no detectable pollution risk. Collectively, the results indicated a low overall bioavailability of HMs in the soil. The results of both analyses indicated that the low risk of environmental contamination by HM accumulation forms was due to the overall weak alkaline nature of the soil in the study area and the low content of HMs in the weak-acid fraction, which made the migration and transformation of HMs weak and the environmental toxicity of HMs low.

#### 3.4.3. Human Health Risk Assessment

(1) Non-carcinogenic risk

**Figure 8:**
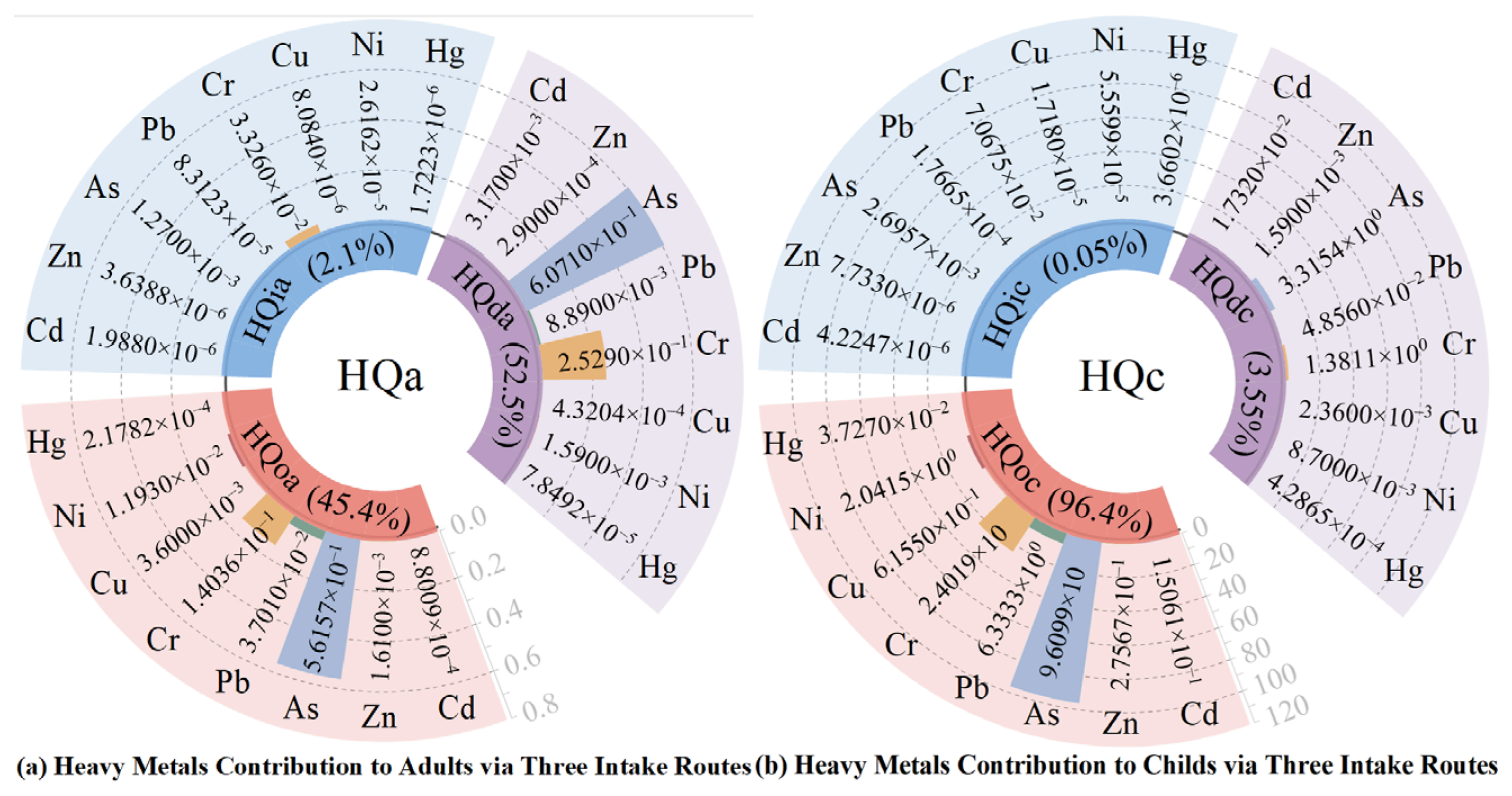
Results of non-carcinogenic risk assessment for adult and child groups. The three color blocks represent the three exposure routes, while the bars represent the corresponding values and are labeled with each heavy metal and the risk index.

A human health risk assessment evaluates non-carcinogenic risks for both adults and children via three primary exposure pathways: ingestion (HQ_o_), inhalation (HQ_i_), and dermal contact (HQ_d_). A Hazard Quotient (HQ) value less than 1 indicates no significant risk, whereas a value exceeding 1 signifies a potential risk [55]. The results of the assessment for the two groups are shown in Figure 8. HQ_oa_, HQ_ia_ and HQ_da_ denote the non-carcinogenic risks for adults via oral, inhalation, and dermal pathways, respectively. The total non-carcinogenic risk in the adults was mainly due to As, Cr and Pb, which contributed 98.57%. The main routes of exposure were the oral and dermal routes, which contributed 45.44% and 52.48%, respectively. HQ_oc_, HQ_ic_ and HQ_dc_ represent the non-carcinogenic risks of the three pathways in children. The main source of the total non-cancer risk in children was As, with a contribution of 74.08%. The oral route was the main exposure route, contributing 96.39%. The non-carcinogenic risk index (HI) for adults was 1.666, indicating a risk; the HI for children was 134.421, which was much greater than 1, indicating a high non-carcinogenic risk.

(2) Carcinogenic risk

The carcinogenic risk assessment for As, Pb, Cr, Cd, and Ni over their entire lifetime was conducted utilizing established carcinogenic slope factors (CSFs), and the results are shown in Table 1. Ro, Ri, and Rd represent the carcinogenic risks via oral ingestion, inhalation, and dermal contact pathways, respectively. Rt denotes the total carcinogenic risk. The carcinogenic risks are categorized into four distinct levels: low risk (10^−6^), medium-low risk (10^−6^~10^−4^), moderate risk (10^−4^~10^−2^) and high risk (10^−2^). Throughout the entire life cycle, carcinogenic risks associated with the three exposure pathways are presented in Table 1, with the oral ingestion pathway demonstrating the highest contribution rate with 99.2%. As constituted the predominant contributor to carcinogenic risk, reaching 99.09%. The total carcinogenic risk was 6.287 × 10^−3^, indicating a moderate risk level within the study area.

(3) Sankey diagram

**Figure 9:**
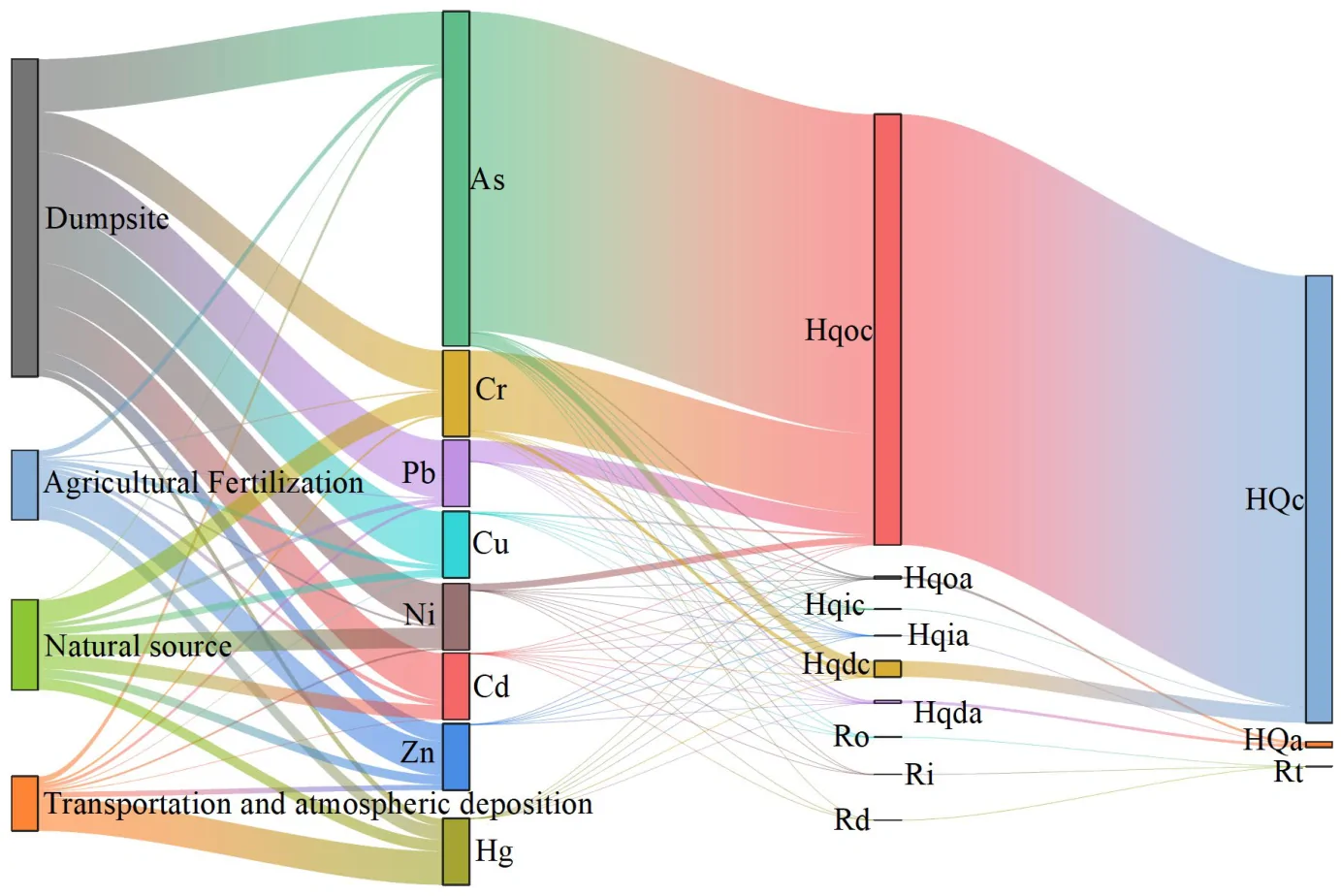
Heavy metal content, sources, and risk assessment of soil in dumpsite.

The results of the APCS-MLR method were used to draw a Sankey diagram, as shown in Figure 9, analyzing the results of the evaluation of sources, species, and human health risks caused by HMs around the dumpsite. The analysis revealed that the dumpsite serves as the primary source of HMs, with As, Cr, and Pb being the main contributors to human health risks. The overall non-carcinogenic risk for both adults and children exceeded the threshold of 1, surpassing acceptable levels. Children are significantly more affected than adults because their bodies are still growing and developing and are more sensitive to the effects of environmental pollution [56]. Over the life cycle, the total carcinogenic risk was at a moderate level.

Although the total concentrations of most HMs were below the risk screening values, and the pollution indices indicated only low to moderate contamination, the human health risk assessment revealed non-carcinogenic and carcinogenic risks exceeding the acceptable thresholds, particularly for children. This discrepancy arises from fundamental differences between the frameworks of pollution indices and health risk assessment. Pollution indices evaluate the degree of environmental enrichment by comparing measured concentrations with background values, thereby reflecting the extent of contamination but not necessarily the potential to induce adverse human health effects.

In contrast, human health risk assessment integrates multiple exposure pathways, exposure duration, and related parameters to more accurately reflect the actual impact of soil HMs on human health. The elevated risk values observed in this study are primarily attributable to three factors: (1) the dominant contribution of As, which, owing to its high oral bioavailability and high carcinogenic slope factor, accounted for 74.08% of the non-carcinogenic risk and 99.09% of the total carcinogenic risk in children; (2) the toxicological relevance of Cr and Ni—hexavalent chromium exhibits a high carcinogenic slope factor via the inhalation pathway, while nickel exerts its effects through dermal and inhalation routes; and (3) the heightened sensitivity of children, who have higher intake rates per unit body weight and developing organ systems, resulting in HI values approximately 80-fold higher than those for adults.

Moreover, carcinogenic risk is characterized by a non-threshold nature, meaning that even chronic low-level exposure over a lifetime can produce measurable health impacts, as health risk is governed by the actual delivered dose, the toxicological potency of each element, and the physiological susceptibility of the exposed population—particularly children. These values should not be interpreted as direct evidence that the metals tested will or will not cause cancer in the exposed population, but rather as screening-level indicators of potential risk that can be used to prioritize remediation efforts and guide risk management decisions.

## 4. Conclusions and Prospects

The Qinghai–Xizang Plateau plays a pivotal role in global atmospheric circulation and water resources. Safeguarding its ecological environment is fundamental to protecting the deep foundations of human civilization. Due to inadequate infrastructure, municipal solid waste disposal in the last century primarily relied on open dumpsites. To evaluate the impact of these dumpsites on the plateau’s ecosystem, this study comprehensively analyzed HM concentrations and speciation in soils surrounding a representative dumpsite in Xizang and conducted source apportionment together with ecological risk assessment.

The results indicate that the concentrations of seven HMs (excluding As) in the soils surrounding the dumpsite exceeded background levels yet remained below the risk-screening values. The relatively high As content was attributed to the region’s unique parent material conditions. Speciation analysis revealed that the residual fraction was dominant, while the weak-acid-extractable fraction was the lowest, indicating low bioavailability and a low potential ecological risk. HM concentrations increased with soil depth, peaking at the 30–50 cm layer. Source apportionment identified the open dumpsite as contributing an average of 59.71% to the total HM concentrations across all analyzed elements, followed by soil parent material (16.97%), agricultural activities (13.09%), and traffic and atmospheric deposition (10.23%). Pollution and ecological risk assessments suggested that the dumpsite posed low to moderate contamination risk and low ecological risk. However, the total non-carcinogenic risk for both adults and children exceeded acceptable levels, primarily driven by As via ingestion and dermal exposure pathways. The total carcinogenic risk was assessed to be at a moderate level.

These findings demonstrate that the dumpsites on the Qinghai–Xizang Plateau exert non-negligible impacts on the surrounding environment and human health. Consequently, it is imperative to promptly implement standard impermeable measures at these sites, ensure non-hazardous waste treatment, and select appropriate vegetation for ecological restoration and site remediation. Future research should incorporate simulated soil column experiments to gain a deeper understanding of the migration patterns of HMs and the associated ecological pollution risks under the unique alpine and high-altitude conditions of the plateau region.

## Figures and Tables

**Table 1:** Carcinogenic risk of HMs in different exposure routes.

Heavy Metal	Ro	Ri	Rd	Rt
As	6.202 × 10^−3^	7.634 × 10^−7^	2.752 × 10^−5^	6.230 × 10^−3^
Pb	2.702 × 10^−5^	-	-	2.702 × 10^−5^
Cr	-	2.190 × 10^−5^	-	2.190 × 10^−5^
Cd	8.207 × 10^−6^	6.647 × 10^−9^	1.949 × 10^−8^	8.233 × 10^−6^
Ni	-	2.481 × 10^−7^	-	2.481 × 10^−7^
Total	6.237 × 10^−3^	2.29 × 10^−5^	2.754 × 10^−5^	6.287 × 10^−3^

## Data Availability

The raw data supporting the conclusions of this article will be made available by the authors on request.

## Supplementary Materials

The following supporting information can be downloaded at https://www.mdpi.com/article/10.3390/toxics14090804/s1, Table S1: Correlation coefficient of potential ecological risk assessment; Table S2: Relevant Information on Parameters; Table S3: Reference dose and carcinogenic slope factor; Table S4: APCS-MLR method for explaining total variance; Table S5: Classification Standards for heavy metals pollution assessment; Table S6: Classification Standards for assessment of the risk of heavy metals speciations; Figure S1: Box plot of soil HMs content in the study area; Figure S2: Proportion chart of different occurrence forms of HMs at each point; Figure S3: Correlation between Soil Physical and Chemical Properties and HMs Content and Forms.
