# Supplementary material for: Contamination Characteristics, Source Apportionment, and Risk Assessment of Heavy Metals in Soil from a Legacy Open Dumpsite on the Qinghai–Xizang Plateau
Source: Toxics. 2026 Sep 10;14(9):804. doi: 10.3390/toxics14090804 (PMC13611465; doi:10.3390/toxics14090804)
Supplement: Supplementary file 1 [file toxics-14-00804-s001.zip › toxics-4471110-supplementary.pdf]

Table S1. Correlation coefficient of potential ecological risk assessment

Standard			Toxic Response (T_r^i)	
E_r^i	Eri<40	Low ecological risk	Cu	5
	40≤Eri<80	Medium ecological risk	Pb	5
	80≤Eri<160	Higher ecological risk	Zn	1
	160≤Eri<320	High ecological risk	Hg	40
	Eri≥320	Serious ecological risk	Cd	30
RI	RI<150	Light ecological risk	Cr	2
	150≤RI<300	Moderate ecological risk	As	10
	300≤RI<600	High ecological risk	Ni	5
	RI≥600	Severe ecological risk		

Table S2. Relevant Information on Parameters

Parameter	Symbol	Unit	Adult	Childre	Notes
Oral intake rate	IngR	$mg·d^{-1}$	100	200	
Soil inhalation rate	InhR	$m^3·d^{-1}$	12.8	7.63	
Exposure frequency	EF	$d·a^{-1}$	350	320	
Exposure delay	ED	a	24	6	
Weight	BW	kg	62	15.9	
Average exposure time	AT	d	8760	2190	noncarcinogenic
		d	26353	26353	carcinogenic
Body surface area	SA	cm^2	2145	1150	
Skin adhesion coefficient	SL	$mg·(cm^2·d)^{-1}$	0.07	0.2	
Skin absorption factor	ABS	dimensionless	As：0.03；other：0.001		
Particle emission factor	PEF	$m^3·kg^{-1}$	$1.36×10^9$	$1.36×10^9$	
End of life average exposure	LADD	$mg·(kg·d)^{-1}$	Calculated value	Calculated value	
Conversion coefficient	CF	$kg·mg^{-1}$	$1×10^{-6}$	$1×10^{-6}$	

Table S3. Reference dose and carcinogenic slope factor

Heavy metal	Reference Dose/mg·(kg·d)$^{-1}$			Skew factor/[mg·(kg·d)$^{-1}$]$^{-1}$		
	RfD_o	RfD_i	RfD_d	SF_o	SF_i	SF_d
Cu	4×10^{-2}	4.02×10^{-2}	1.2×10^{-2}	--	--	--
Pb	3.5×10^{-3}	3.52×10^{-3}	5.25×10^{-4}	8.5×10^{-3}	--	--
Zn	3×10^{-1}	3×10^{-1}	6×10^{-2}	--	--	--
Hg	3×10^{-4}	$8.57\times10_{5}$	3×10^{-5}	--	--	--
Ni	2×10^{-2}	2.06×10^{-2}	5.4×10^{-3}		8.4×10^{-1}	
Cd	1×10^{-3}	1×10^{-3}	1×10^{-5}	0.38	6.1	6.1
Cr	3×10^{-3}	2.86×10^{-5}	6×10^{-5}	--	42	--
As	3×10^{-4}	3×10^{-4}	3×10^{-4}	1.5	3.66	1.5

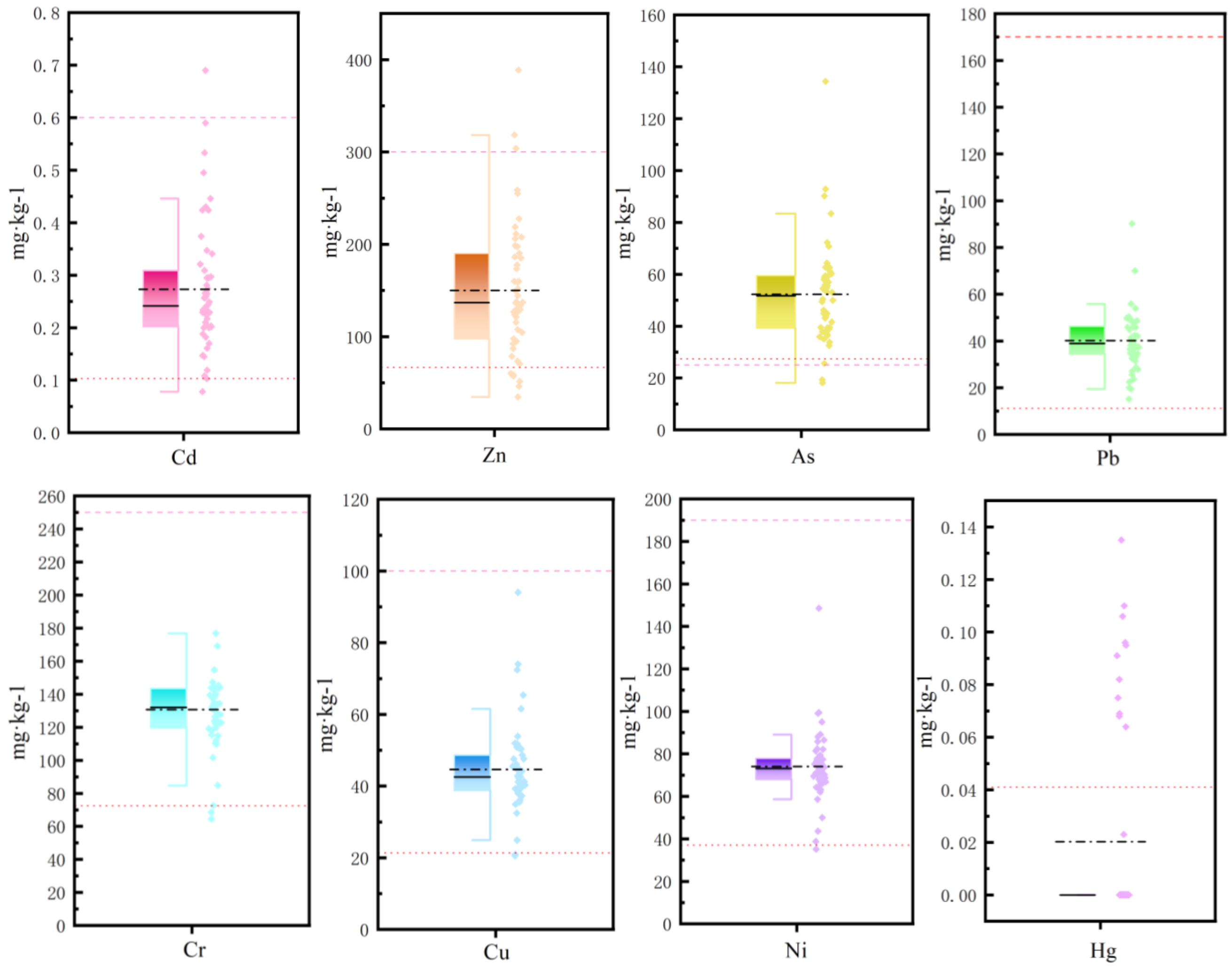

Figure S1. Box plot of soil HMs content in the study area

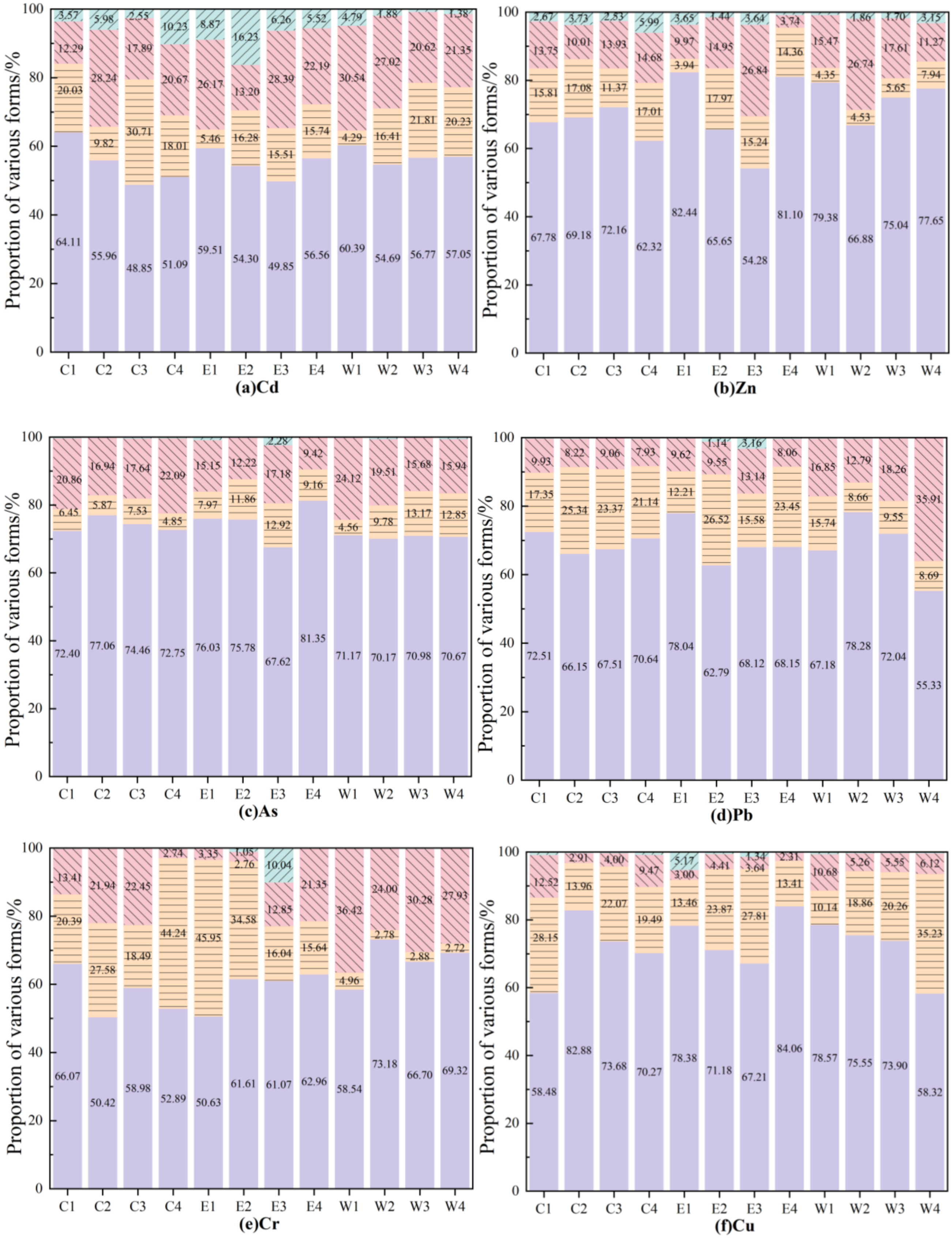

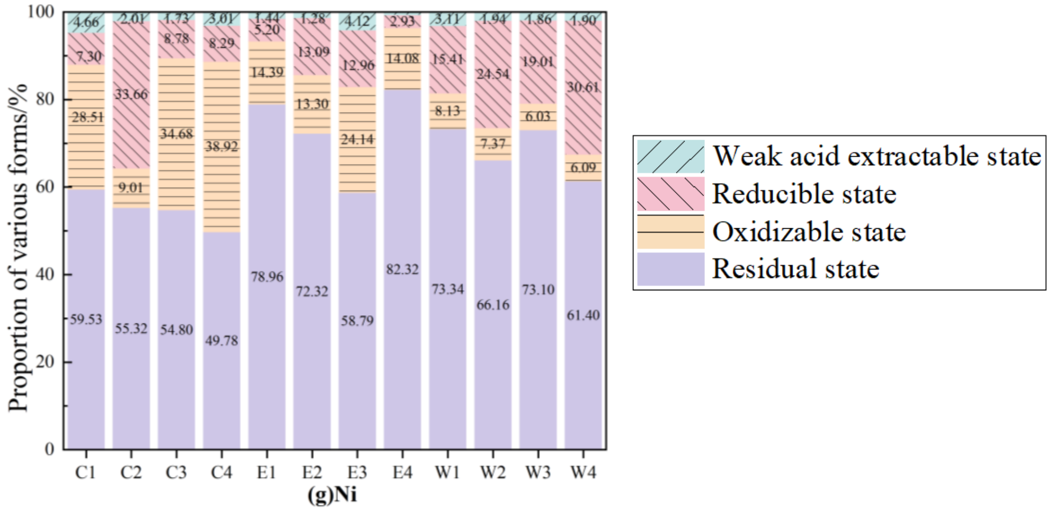

Figure S2. Proportion chart of different occurrence forms of HMs at each point

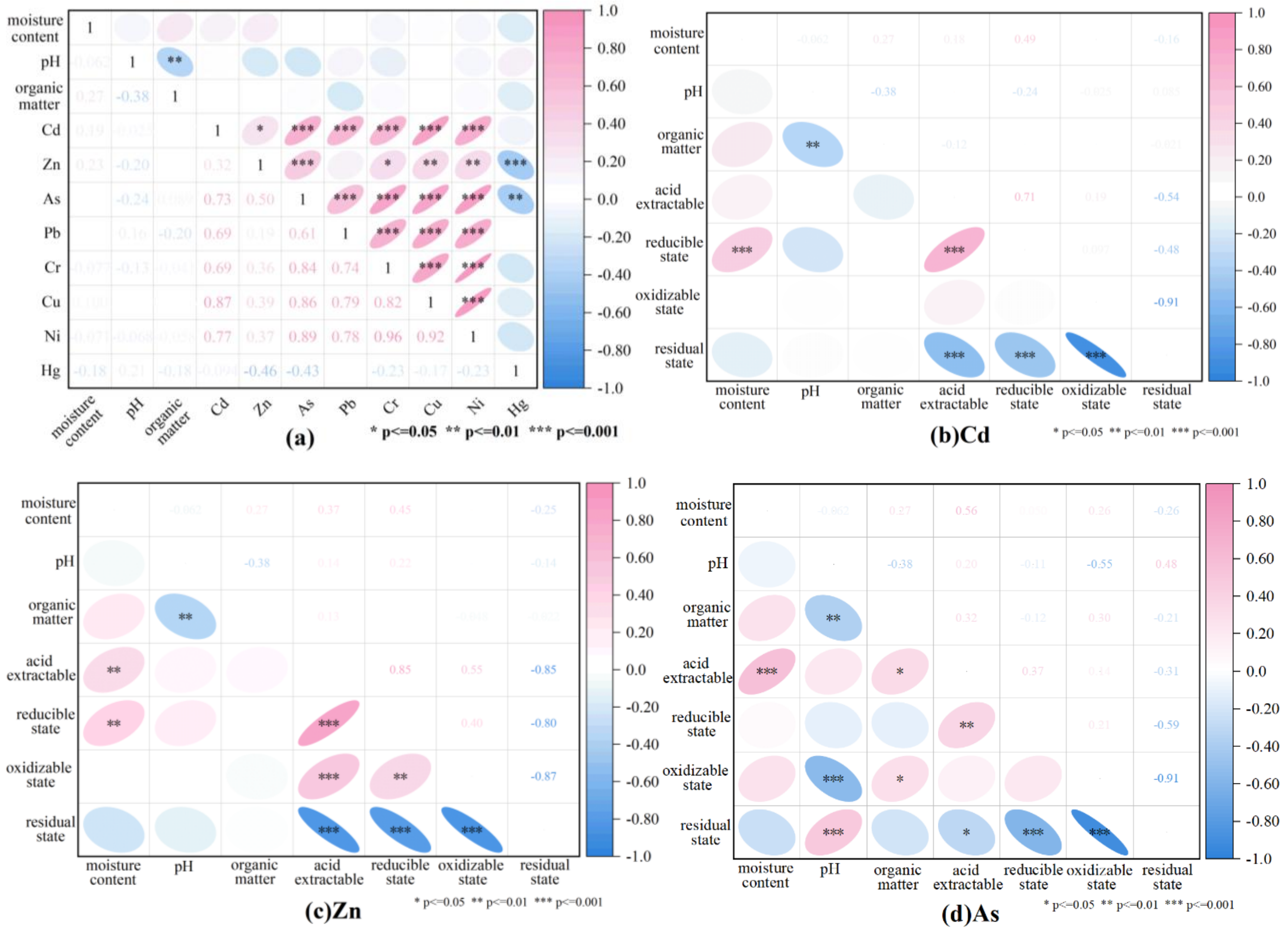

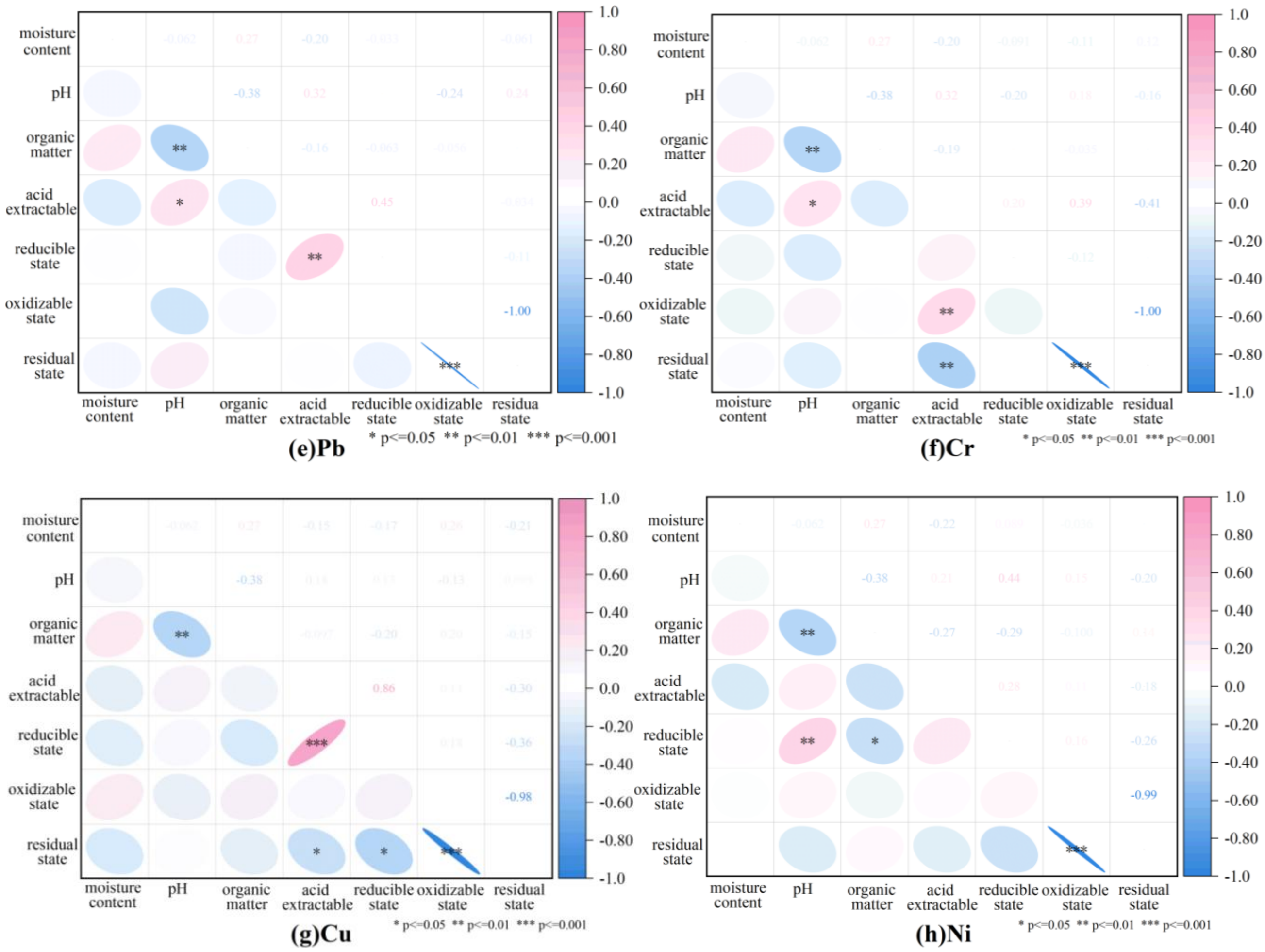

Figure S3. Correlation between Soil Physical and Chemical Properties and HMs Content and Forms

Table S4. APCS-MLR method for explaining total variance

Explanation of Total Variance						
Component	Initial eigenvalue			Extract the sum of squared loads		
	Total	Variance percentage	Accure %	Total	Variance percentage	Accure %
1	5.248	65.601	65.601	5.248	65.601	65.601
2	1.396	17.448	83.050	1.396	17.448	83.050
3	0.547	6.840	89.890	0.547	6.840	89.890
4	0.355	4.437	94.327	Sum of squared rotational loads		
5	0.244	3.052	97.379	Total	Variance percentage	Accure %
6	0.119	1.485	98.864	4.840	60.500	60.500
7	0.072	0.897	99.761	1.246	15.581	76.080
8	0.019	0.239	100.000	1.105	13.809	89.890

Extraction method: Principal Component Analysis.

Table S5. Classification Standards for heavy metals pollution assessment

Single factor pollution index		Geo-accumulation index		comprehensive pollution load index	
Index Range	Pollution levels	Index Range	Pollution levels	Index Range	Pollution levels
$P_i \leq 1$	no pollution	$I_{geo} < 0$	no pollution	PLI ≤ 1	no pollution
$1 < P_i \leq 2$	slight pollution	$0 < I_{geo} < 1$	slight pollution	1 < PLI ≤ 2	slight pollution
$2 < P_i \leq 3$	moderate pollution	$1 < I_{geo} < 2$	moderate pollution	2 < PLI ≤ 3	moderate pollution
$P_i > 3$	severe pollution	$2 < I_{geo} < 3$	moderate pollution	3 < PLI ≤ 5	severe pollution
				PLI > 5	heavy pollution

Table S6. Classification Standards for assessment of the risk of heavy metals speciations

Risk assessment coding		Khan Risk Synthesis Potential	
Index Range	Pollution levels	Index Range	Pollution levels
RAC < 1%	no risk	KRSP < 100%	no pollution
1% ⩽ RAC < 10%	low risk	100% ⩽ KRSP < 200%	slight pollution
10% ⩽ RAC < 30%	moderate risk	200% ⩽ KRSP < 300%	moderate pollution
30% ⩽ RAC < 50%	high risk	KRSP ⩾ 300%	high pollution
RAC ⩾ 50%	severe risk		
